# Paediatric HeartMate 3™, the Uneventful 22-Month Journey to Transplantation of a 14-Year-Old-Patient—Time for Prolonged LVAD Support in Children?

**DOI:** 10.3390/jcdd11090288

**Published:** 2024-09-18

**Authors:** Clemens Haselmann, Sonja Budäus, Michael Zellner, Robert Cesnjevar, Martin Schweiger

**Affiliations:** 1 Division of Cardiac Surgery, University Children’s Hospital Zurich, 8032-Zurich, Switzerland; 2 Division of Paediatric Cardiology, University Children’s Hospital Zurich, 8032-Zurich, Switzerland; 3 Department of Diagnostic Imaging, University Children’s Hospital Zurich, 8032-Zurich, Switzerland

**Keywords:** LVAD, cardiac transplantation, DCM, paediatric cardiac surgery

## Abstract

We report on a 14-year-old patient who was supported for nearly two years with an ic-LVAD and managed to complete his journey to transplantation without a single complication. Although mechanical assist device support is available for children up to 20 kg in body weight, availability is limited to paracorporeal devices. Intracorporal (ic) left ventricular assist devices (LVADs) for infants in the suitable weight class are a viable option as a bridge-to-transplant, where they make up more than 50% of transplant candidates in their category. A teenager with 59 kg body weight was newly diagnosed with DCM and listed for heart transplantation. After initially being on VA-ECMO, an Abbott HeartMate 3 LVAD with postoperative temporary RVAD support was initialised. RV-support was maintained for 10 days. The further postoperative course was uneventful, and he was discharged on day 98. He was seen regularly in the outpatient department and integrated into school routine again, following the extensive training of his classmates and the responsible school staff. After a total of 672 days on support, he was successfully transplanted. There were no unplanned admissions, thrombotic nor bleeding events, as well as no driveline infection, even though the patient participated in sport classes at school.

## 1. Introduction

Dilated cardiomyopathy is a common late diagnosis in paediatric and adolescent cardiac patients, with a reported annual incidence of about 0.57 up to 6.95 cases per 100,000 patients [1,2]. The established treatment for a paediatric patient diagnosed with dilated cardiomyopathy (DCM) and severe heart failure awaiting transplantation is bridging via the Berlin Heart EXCOR System (Berlin Heart, Berlin, Germany). To facilitate mobility and allow for reintegration into their social life, a push towards intracorporal devices has been made for younger patients. The HeartMate 3 (Abbott Laboratories, Abbott Park, IL, USA) Left Ventricular Assist Device (LVAD) system is currently the only available option for teenagers. While waiting times in North America for young patients are low, in Europe, only 1/3 of paediatric patients listed for a VAD are transplanted after 6 months [3], despite a markedly older median donor age [4]. Overall, within the adolescent group, patients with DCM present the majority of heart transplant patients, despite a slightly declining trend [5].

## 2. Case

We report on the case of a now 16-year-old boy, diagnosed with DCM at age 14. Upon the initial presentation, an echo revealed a moderate-to-severe mitral regurgitation, mild-to-moderate tricuspid insufficiency, and a left ventricular ejection fraction (LVEF) of only 20%, with further laboratory signs of end-stage heart failure. A nasal swab for rhino/enterovirus proved to be positive, explaining his sudden deterioration. He was admitted straight to the PICU with a then body weight of 59.2 kg (BMI 20.7 kg/m²).

Genetic classification was initiated, presenting a mutation of the Phospholamban (PLN) gene, which is known to be associated with DCM, although this was not described as a point mutation R14del, the most common alteration on the PLN gene [6,7,8]. Accordingly, the electrocardiogram showed sinus bradycardia without any additional rhythmic abnormalities. Cardiac catheterisation presented a normal coronary configuration but revealed an elevated pulmonary resistance (3.1 WU/m^2^) and pulmonary hypertension. Endomyocardial biopsy gave no additional hint of an infectious etiology.

Further decompensation after an initial treatment for 2 weeks with inotropes led to intubation and, on the following day, VA-ECMO treatment via inguinal access had to be initialised (INTERMACS Profile 2).

After 8 days on ECMO, with a body weight of 51.1 kg, he underwent an LVAD placement while parallelly being listed for cardiac transplantation with the blood type O+. A HeartMate 3 LVAD was placed in the typical fashion via apical and aortic cannulation. After a good initial ramp-up, the right ventricular system showed, despite the adequate inotropic support, insufficient compliance. This led to the temporary EMCO support of the right heart with cannulation of the pulmonary artery via an 10mm Dacron graft and the previously used femoral vein. The flows before transfer to the ICU were 3.2 L/min on the RVAD-ECMO and 2.6–2.8 L/min at 4800 rpm on the LVAD under moderate catecholamine support.

Treated on postoperative day (POD) 1 for a pericardial tamponade, the RVAD system was removed after 10 days and the patient was extubated on day 13. The initial anticoagulation management was ASA and unfractionated heparin, with an anti-factor Xa range of 0.3–0.5 IU/mL. After maintaining good blood pressure control, he was transferred to the paediatric cardiology ward on day 35. Approximately 73 days after LVAD implantation and a combined 98 days post-presentation at the A&E, he was discharged home on phenprocoumon, with a target INR range of 2–3, and ASA 100mg once daily, while the LVAD was maintained at 4800 rpm (rounds per minute). To prepare for his discharge, his family and teachers were instructed on the LVAD use and emergency handling. The patient and his family received extensive training lessons on the handling and troubleshooting of the device as well as anticoagulation issues. Prior to discharge, additional training of the local Emergency Medical Services was organised as well as the primary care physician. The patient’s family resided within a reasonable driving distance to our institution.

**Figure 1:**
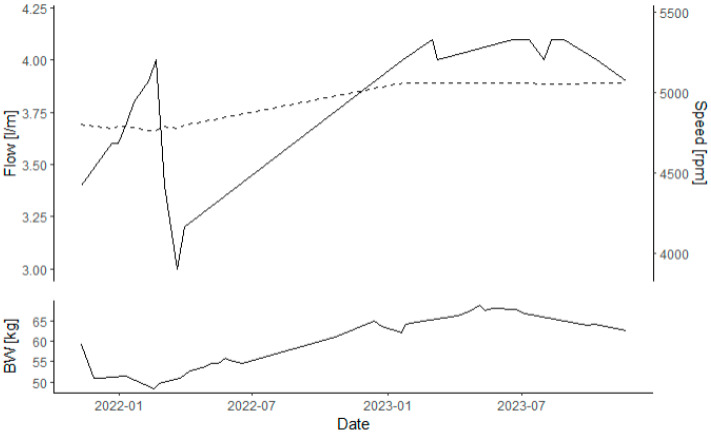
Graph presenting the course of flow (top, solid line) and set speed (top, dashed line) on the LVAD as well es body weight (buttom) during the support period.

Outpatient monitoring was scheduled once weekly, with echocardiography and a laboratory assessment. After three months, we could analyse one Low Flow event on the device at around 6 am, without any further adjustment necessary. While initially home schooled, he was reintegrated back into his daily school routine after further the training of his teachers and providing instructions to his classmates after 3 months. He maintained good INR control over the whole period and stayed without syncope or palpitations, although insufficient water intake led to some episodes of dizziness. Figure 1 shows his weight history over the treatment period, which was stabilised medically with additional torasemide.

Over the first winter period, a revaluation in the cath lab presented a mean PA pressure of 15 mmHg, with a slightly elevated pulmonary resistance. He was stable at the time at around 60–62 kg and with a restricted oral intake of two litres per day. The left ventricle showed signs of dilatation, and an echo and cath confirmed no output of the aortic valve; therefore, we increased the LVAD to 5100 rpm. This was the only outpatient adaption necessary on the HM3.

**Figure 2:**
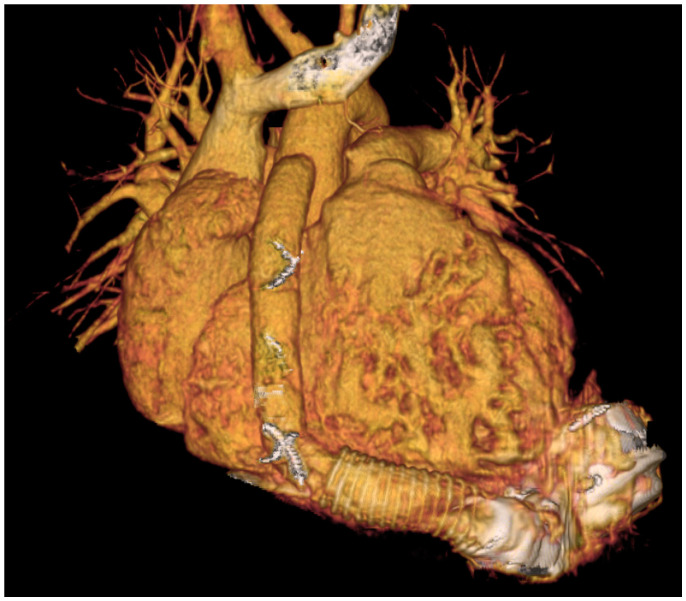
CT thorax anterior oblique virtual 3D rendering showing LVAD in place.

During ongoing support, we reconfirmed good positioning of the LVAD as well as the freedom of signs for thrombus formation via CT scan, as seen on Figure 2.

After a total of 672 days on the waiting list, we successfully transplanted the patient, with an initial immunosuppression schema consisting of ATG and prednisolone. Extubated on day 5 and on hemofiltration for 10 days post-surgery, he was sent to the ward on day 16. With excellent graft function and no signs of rejection, we could discharge him home after 36 days on tacrolimus, mycophenolate mofetil, and prednisolone.

## 3. Discussion

Within this case report, we present the uneventful course of intra-corporeal LVAD support in a teenager for almost 2 years, Box 1 summarizes the key takeaway points.

Box 1 **Key takeaway points.**

- Children and adolescents will benefit when brought into their natural environment (home, school, children’s playground) as soon as possible to improve quality of life.
- Anticoagulation can be maintained on an oral basis and through somatic growth with good compliance, even in adolescents.
- Long-term intra-corporeal LVAD therapy in adolescents is possible with very few complications and might open the door to prolonged LVAD support in this patient population.

The successful discharge of children on intra-corporeal LVAD support has previously been reported [9] to minimize complications and enhance quality of life. Patient and family training helps maintain the daily care of the system and driveline, including sterile dressing and wound care, and also reduces absences from school; these remain crucial points to achieve optimal long-term outcomes. 

Anticoagulation remains another important issue. In this case, a well-maintained INR range showed good compliance and resulted in no bleeding or embolic events despite the patient’s regular adolescent social behaviour, including school visits, social interactions, or sport activities. It should be mentioned that this patient was on double anticoagulation while there was a trend towards single anticoagulation only. 

We also faced no problems due to somatic growth in the teenager. Previous authors have shown the importance of outflow tract position and angle as they correlate with thrombus formation as well as signal an aneurysm formation at the outflow tract anastomosis [10,11]. Due to these factors, our institutional policy is to perform periodical routine CT imaging. None of these possible complications were observed despite the increase in body weight from 49 to 69 kg and 5 cm in height. This goes in accordance with the observation that only once device setting had to be changed. 

There are reports from The ACTION Network by O’Connor et al., who reported their experience for the HeartMate 3 for a congenital heart patient who weighed as low as 19.1 kg. The median and peak duration was 40 and 310 days [12]. While a majority of the patients with intra-corporeal LVAD support have been reported from the U.S., the waiting times for a suitable organ seems to be much higher in Europe. Cao et al. from Italy recently published their experience on four paediatric HM3 devices with a calculated median time to transplant of 206 days [13]. If the paediatric MCS community confirms uneventful intra-corporeal LVAD courses like ours in larger trials, this might lead to a paradigm change for children. We know that survival after cardiac retransplantation is relatively low compared to the first transplant [14]. Thus far, the need for destination therapy as long-term or permanent support in children is very rare. The most recent data by the ISHLT have shown a median freedom from death or retransplantation of 14.2 years for paediatric heart transplant patients [15]. We might be able to develop a new treatment strategy for children in need of transplantation. We should think about implanting a durable MCS device before the first transplant, thus gaining another two to five years of life beforehand. In adults, trials like the ELEVATE registry and MOMENTUM3 showed reduced rates of bleeding and stroke as well as lower pump thrombosis rate and the overall survival after 5 years of 63.3% and 58.4% [16,17]. Although we have not reached that point for children and adolescents yet, we may need to discuss different strategies for our young patients. 

## 4. Conclusions

Our vision for optimal intra-corporeal LVAD support is to minimize the social and personal impacts on the patient due to growing up as well as provide him and everyone involved in his care with lives that are as unimpactful as possible. 

## Data Availability

Data generated for this report is due to reasons of patient privacy not available.
